# Structural and Functional Characterization of DUF1471 Domains of *Salmonella* Proteins SrfN, YdgH/SssB, and YahO

**DOI:** 10.1371/journal.pone.0101787

**Published:** 2014-07-10

**Authors:** Alexander Eletsky, Karolina Michalska, Scott Houliston, Qi Zhang, Michael D. Daily, Xiaohui Xu, Hong Cui, Adelinda Yee, Alexander Lemak, Bin Wu, Maite Garcia, Meagan C. Burnet, Kristen M. Meyer, Uma K. Aryal, Octavio Sanchez, Charles Ansong, Rong Xiao, Thomas B. Acton, Joshua N. Adkins, Gaetano T. Montelione, Andrzej Joachimiak, Cheryl H. Arrowsmith, Alexei Savchenko, Thomas Szyperski, John R. Cort

**Affiliations:** 1 Department of Chemistry, The State University of New York at Buffalo, Buffalo, New York, United States of America; 2 Structural Biology Center, Biosciences Division, Argonne National Laboratory, Argonne, Illinois, United States of America; 3 Princess Margaret Cancer Centre and Department of Medical Biophysics, University of Toronto, Toronto, Ontario, Canada; 4 Fundamental and Computational Sciences Directorate, Pacific Northwest National Laboratory, Richland, Washington, United States of America; 5 Center for Advanced Biotechnology and Medicine, Department of Molecular Biology and Biochemistry, Rutgers, The State University of New Jersey, Piscataway, New Jersey, United States of America; 6 Department of Biochemistry and Molecular Biology, Robert Wood Johnson Medical School, Rutgers, The State University of New Jersey, Piscataway, New Jersey, United States of America; 7 Department of Chemical Engineering and Applied Chemistry, Banting and Best Department of Medical Research, University of Toronto, Toronto, Ontario, Canada; 8 Midwest Center for Structural Genomics, Biosciences Division, Argonne National Laboratory, Argonne, Illinois, United States of America; 9 Northeast Structural Genomics Consortium; MRC National Institute for Medical Research, United Kingdom

## Abstract

Bacterial species in the Enterobacteriaceae typically contain multiple paralogues of a small domain of unknown function (DUF1471) from a family of conserved proteins also known as YhcN or BhsA/McbA. Proteins containing DUF1471 may have a single or three copies of this domain. Representatives of this family have been demonstrated to play roles in several cellular processes including stress response, biofilm formation, and pathogenesis. We have conducted NMR and X-ray crystallographic studies of four DUF1471 domains from *Salmonella* representing three different paralogous DUF1471 subfamilies: SrfN, YahO, and SssB/YdgH (two of its three DUF1471 domains: the N-terminal domain I (residues 21–91), and the C-terminal domain III (residues 244–314)). Notably, SrfN has been shown to have a role in intracellular infection by *Salmonella* Typhimurium. These domains share less than 35% pairwise sequence identity. Structures of all four domains show a mixed α+β fold that is most similar to that of bacterial lipoprotein RcsF. However, all four DUF1471 sequences lack the redox sensitive cysteine residues essential for RcsF activity in a phospho-relay pathway, suggesting that DUF1471 domains perform a different function(s). SrfN forms a dimer in contrast to YahO and SssB domains I and III, which are monomers in solution. A putative binding site for oxyanions such as phosphate and sulfate was identified in SrfN, and an interaction between the SrfN dimer and sulfated polysaccharides was demonstrated, suggesting a direct role for this DUF1471 domain at the host-pathogen interface.

## Introduction

**Figure 1:**
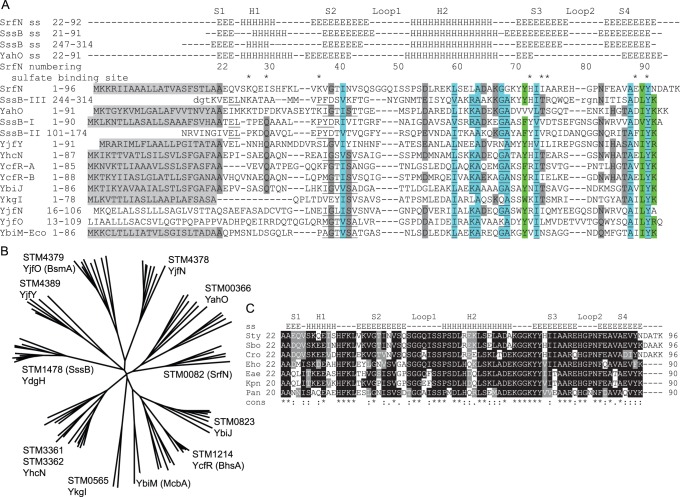
DUF 1471 sequences. **A**: Multiple sequence alignment of DUF1471 paralogues from *S.* Typhimurium, as well as *E. coli* YbiM, for which there is no close homolog in *Salmonella*. Alignment of SrfN, YahO, SssB-I and SssB-III is structure-based over the entire structured sequence (SrfN residues 22–91), other alignments are sequence-based and are between core regions only (SrfN residues 35–91) because sequence identity to SrfN residues 22–38 is low and alignments in this region are uncertain. Secondary structure in SrfN, YahO, SssB-I, and SssB-III is indicated above: E = extended (β-sheet) structure, H = helix. The core residues of the sulfate-binding motif in SrfN are indicated with asterisks. Conserved sequence motifs identified by Rudd [1] are underlined. Other conserved residues are highlighted in green or dark grey. Two notable loop regions in the structure are also indicated. SrfN and YahO both have C-terminal tag sequences LEHHHHHH that are not shown. Light grey highlighted portions indicate likely signal sequences for periplasmic localization that are known or likely to be cleaved by a signal peptidase. In the case of SrfN and YahO, the signal sequence was proven experimentally to be cleaved during heterologous expression in *E. coli*. Inter-domain regions of SssB are not shown. Lower case letters in SssB-III (C-terminal domain) indicate residues with missing electron density in the X-ray structure. Highly conserved residues are indicated by highlighting (blue = hydrophobic, green = polar), somewhat conserved residues are indicated with grey highlighting. The following sequences are listed (*S.* Typhimurium LT2 locus and UniProt/TrEMBL numbers in parentheses): SrfN (STM0082/Q7CR88), YjfY (STM4389/Q8ZK84), YhcN (STM3361/Q8ZLP6), YcfR seq. I (STM1214/Q8ZQ03), YcfR seq. II (STM3362/Q7CPN0), YahO (STM0366/Q7CR49), YbiJ (STM0823/Q7CQW3), YkgI (STM0565/Q7CR04), YjfO (STM4379/Q8ZK92), YjfN (STM4378/Q8ZK93), SssB (STM1478/Q8ZPL1), YbiM/McbA (*E. coli*, P0AAX6). **B**: Unrooted phylogenetic tree (phylogram) constructed from ten diverse genera from the Enterobacteriaceae. Major branches containing *Salmonella* and *E. coli* subfamily members are indicated. **C**: Multiple sequence alignment of SrfN homologues: a subfamily of DUF1471 proteins. For each sequence and abbreviated organism name listed, the full genus and species name, protein/ORF name, database accession number, and similarity to SrfN, excluding the signal sequence, are as follows: Sty, *Salmonella enterica* Typhimurium, STM0082 (SrfN), NP_459087 and many other *Salmonella* strains; Sbo, *Salmonella bongori*, SBG_0068, YP_004728986 (93%); Cro, *Citrobacter rodentium*, ROD_12311, YP_003364817 (80%); Eho, *Enterobacter hormaechei*, HMPREF9086_0329, ZP_08496071 (65%); Eae, *Enterobacter aerogenes* EAE_13230, YP_004592839 (70%); Kpn, *Klebsiella pneumonia*, KPK_4095, YP_002239898 (68%); Pan, *Pantoea* sp., Pat9b_3745, YP_004117591 (61%). Notes: Other *Salmonella, Klebsiella,* and *Enterobacter* species and strains contain identical or nearly identical sequences to the representatives shown here. However, some *Pantoea* species do not contain homologues that fall within this DUF1471 subfamily.

Many of the basic features of sequences in the family of conserved proteins from Enterobacteriaceae now known as DUF1471 (PF07338, alternatively, the YhcN or BhsA/McbA family) were recognized [1] soon after release of the first *E. coli* genome sequence. These proteins are characteristically small, single domain proteins of around 90 residues; though one subfamily has three repeated DUF1471 domains. Most DUF1471 sequences contain apparent signal sequences that target them to the periplasm. The DUF1471 family includes several hundred members, all occurring exclusively in the Enterobacteriaceae, such as *Escherichia, Salmonella, Yersinia,* and *Shigella*, among others. By sequence analysis the DUF1471 family members can be divided into around twelve paralogous subfamilies with high (greater than 50%) intra- and low (20–40%) inter-subfamily sequence identity and few residues that are conserved across all DUF1471 subfamilies (Fig. 1). Not all paralogous subfamilies are represented in a given organism with DUF1471 domains. For example, there are eleven paralogues each in *Salmonella* and *Klebsiella pneumoniae*–the largest repertoire of DUF1471 proteins found in bacterial genomes–whereas there are ten paralogues in *E. coli*, and five in *Yersinia pestis*. *Salmonella* lacks a homologue of *E. coli* YbiM; the most similar *Salmonella* sequence to *E. coli* YbiM is YcfR (47% sequence identity), which is rather clearly the homologue of *E. coli* YcfR, with 92% identity. *S.* Typhimurium LT2 and many other strains also have a second *ycfR*-like gene (∼55% identity at the protein level) elsewhere in their genomes that is the more similar or the two (90% identity) to the single YcfR from *E. coli*. Such characteristics suggest expansion of the ancestral family either by extensive lateral gene transfer, or through repeated gene duplication followed by sequence divergence and functional specialization, or by a combination of these. Consistent with a pattern of family expansion by gene duplication and functional divergence, we note the aforementioned instance of multiple DUF1471 domains in one polypeptide (YdgH, also known as SssB [2]), as well as at least two instances of adjacent DUF1471 genes in a genome; for example in *Salmonella*, the *yjfN* and *yjfO* genes are adjacent as are *yhcN* and one of its *ycfR*-like genes. To begin understanding the function of DUF1471 proteins, we determined three-dimensional structures of three representative DUF1471 family proteins from *Salmonella*: SrfN, YahO, and YdgH/SssB domains I and III.

The SrfN (STM0082 in *S*. Typhimurium LT2) protein is one of a few DUF1471 paralogues for which limited experimental data has provided some clues to the function. SrfN has close homologues in *Enterobacter, Klebsiella, Citrobacter,* and *Pantoea* species but not in *E. coli* (Fig. 1). In *Salmonella* SrfN expression is controlled by the SPI-2-encoded regulator SsrB, suggesting its involvement in *Salmonella* pathogenicity, and *srfN* inactivation mutants display attenuated virulence in mice [3], [4]. SrfN has been recently characterized as an effector protein secreted into host cells via outer membrane vesicles [4], [5], rather than Type III secretion [2], though the host factors with which it interacts are not known.

SssB (STM1478, also known as YdgH) is another *Salmonella* DUF1471 paralogue implicated in pathogenesis. Like SrfN, it appears to be secreted into macrophages by a mechanism independent of Type III secretion, and a mutant in which the *ydgH/sssB* gene was inactivated was significantly attenuated for virulence in a mouse model one day post-infection, but recovered by day four to wild-type level [2]. This suggests greater importance for acute rather than systemic infection. SssB and its homologues contain three DUF1471 domains (I, II, and III). Predicted lower-complexity regions of approximately 10 and 70 residues separate the first and second and the second and third domains, respectively. Each of the three SssB domains has high similarity to the equivalently positioned domain in SssB sequences from other species, with somewhat lower similarity to SssB domains at different positions (<35% identity), and even lower similarity to other DUF1471 sequences apart from SssB.

The *Salmonella* protein YahO (STM0366) from DUF1471 has not been characterized prior to the work presented here. YahO occurs in *Salmonella, Escherichia*, and numerous other genera from the Enterobacteriaceae. The *yahO* gene was identified as one of several regulated by the alternative sigma factor σ^S^ (RpoS), which is required for *Salmonella* virulence in mice [6]. Furthermore, expression of *yahO* appears to be up-regulated in low pH media that may mimic conditions in the *Salmonella*-containing vesicle [7].

Characterization of several other DUF1471 proteins using genetic and transcriptomic approaches appears to show roles in stress-response and resistance and/or biofilm formation, although the molecular mechanism(s) of these functions remain unknown. A report by Zhang and others shows that *E. coli* YcfR (renamed BhsA) is induced in biofilms and by several stress conditions, and that *ycfR/bhsA* deletion mutants show greater sensitivity to various types of stress, suggesting a general role for YcfR/BhsA in controlling biofilm formation [8]. YcfR/BhsA was found to be the most up-regulated (>10-fold) *E. coli* protein following chlorine treatment [9], and was dramatically up-regulated upon exposure of *E. coli* to lettuce leaf lysate [10]. In *Salmonella*, YcfR/BhsA was shown to be up-regulated >2-fold during chlorine-induced oxidative stress [11], and recently a role for YcfR/BhsA in promoting the attachment of *S*. Typhimurium to glass, polystyrene, and the surfaces of spinach leaves and tomato fruit was demonstrated in a *ycfR/bhsA* deletion mutant [12]. Similar roles for YcfR/BhsA related to attachment to vegetables have been shown in *E. coli* as well [13]. Another study demonstrates involvement of *E. coli* DUF1471 protein YbiM (renamed McbA) in inhibiting biofilm formation and overproducing colanic acid, a polysaccharide that in excess causes mucoidy, by an unknown mechanism that is controlled by the MqsR-regulated transcription factor YncC (also known as McbR) [14]. *Salmonella* does not have a close YbiM/McbA homologue. The DUF1471 protein YjfO (renamed BsmA) also influences biofilm formation in *E. coli* in response to stress, again by an unknown mechanism [15]. Finally, yet another DUF1471 protein, YhcN, was found to be up-regulated in chlorine stress in *S.* Typhimurium, but not *S.* Enteritidis [11]. In *E. coli*, YhcN is linked to oxidative and acid stress responses and to biofilm formation [16]. The DUF1471 subfamilies represented by YbiJ, YjfN, YjfY, and YkgI, remain uncharacterized. Many of the phenomenological observations associated with DUF1471 genes are consistent with the suggestion of Rudd and coworkers [1] that proteins in this family may have “a possible species-specific function, such as self-identification or colony organization using cell-cell contacts or intercellular signaling.”

To approach DUF1471 protein function from a structural standpoint, members of the family were selected for structural characterization by the Northeast Structural Genomics Consortium (NESG), according to criteria guiding the second phase of the Protein Structure Initiative (PSI-2) for maximizing structural coverage and leverage of protein families [17]. The structures of four DUF1471 domains from three *Salmonella* proteins were determined. Structures of SrfN, YahO, and the N-terminal domain (I) of SssB were determined by solution state NMR spectroscopy by NESG (NESG target IDs StR106, StR109, and StR147A). The structure of the C-terminal domain (III) of SssB was determined by X-ray crystallography by the Midwest Center for Structural Genomics (MCSG); target ID APC101565. Here we present structural aspects of these DUF1471 family members through comparative analysis.

## Results and Discussion

The atomic coordinates for the four structures of DUF1471 proteins or domains from *Salmonella* described here have been deposited into the PDB with accession codes 2MA8 (SrfN), 2MA4 (YahO), 2M2J (SssB-I), and 4EVU (SssB-III). The structure factors for 4EVU were also deposited with the coordinates. The chemical shifts for the NMR structures have been deposited in BioMagResBank with accession codes 15090 (SrfN), 19327(YahO), and 18917 (SssB-I).

### Structure Determination of SrfN, YahO, and SssB-I by NMR spectroscopy

Structures of *Salmonella* SrfN, YahO, and SssB-I proteins from the DUF1471 protein family were determined with solution state NMR spectroscopy by the Northeast Structural Genomics Consortium (targets StR109, StR106, and StR147A respectively). Structure statistics for the NMR ensembles are shown in Table 1. The N-termini of these and most other DUF1471 sequences contain predicted signal sequences of around 20 residues for export to the periplasm via a Sec-dependent pathway. Although the constructs used for expression of SrfN and YahO coded for expression of the full length sequences, no chemical shift assignments could be determined for the first 21 residues.

**Table 1:** Summary of NMR and structural statistics^a^ for *Salmonella* SrfN, YahO, and SssB-I.

	SrfN		YahO		SssB-I	
**Completeness of resonance assignments** b						
Backbone (%)	100.0		100.0		92.1	
Side chain (%)	97.6		99.1		89.3	
Stereospecific methyl (%)	100.0		100.0		0.0	
**Conformationally-restricting restraints**						
Distance restraints						
Total	1876		1622		1212	
intra-residue (*i* = *j*)	264		269		157	
sequential (|*i − j*| = 1)	362		411		348	
medium range (1< |*i* – *j*| <5)	378		335		207	
long range (|*i – j*| ≥5)	872		607		500	
intermolecular	34		–		–	
Dihedral angle restraints	182		100		78	
Hydrogen bond restraints	78		60		0	
Total number of restricting restraints	2210		1782		1290	
Restricting NOE restraints per residue	14.8		24.4		19.0	
Long-range restraints per residue	6.4		8.7		7.4	
**Residual restraint violations** c						
Average distance restraint violations per structure^c^						
0.1–0.2 Å	0.1		8		4.3	
0.2–0.5 Å	0		1.25		0.4	
>0.5 Å	0		0		0	
RMS violation per restraint/max viol. (Å)	0.00/0.15		0.02/0.38		0.01/0.34	
Average dihedral restraint violations per structure						
1–10°	0		6.75		3.1	
>10°	0		0		0	
RMS viol. per restraint/max viol. (°)	0.04/0.50		0.66/6.90		0.47/4.20	
**RMSD (ordered/all) from average coordinates** (Å)^d^^,^ e						
	chain A	A&B				
backbone atoms (C,C^α^,N)	0.5/0.6	0.7/0.8	0.4/0.7		0.5/0.8	
heavy atoms	0.9/1.1	1.0/1.2	0.8/1.1		0.9/1.3	
**RPF Recall/Precision/DP-score** [50]	0.87/0.92/0.75		0.98/0.93/0.88		0.96/0.94/0.86	
**MolProbity ** [62] ** Ramachandran statistics**(ordered/all)^d^^,^ e						
most favored regions (%)	96.3/94.2		98.1/92.1		96.0/94.5	
allowed regions (%)	3.7/5.5		1.7/5.9		4.0/5.4	
disallowed regions (%)	0.0/0.3		0.3/1.2		0.0/0.1	
**Global quality scores** (raw/*Z*-score)	raw	Z	raw	Z	raw	Z
Verify3D	0.33	−2.09	0.40	−0.96	0.37	−1.44
ProsaII	0.45	−0.83	0.61	−0.17	0.56	−0.37
Procheck G-factor (phi-psi)^d^	−0.31	−0.90	−0.09	−0.04	−0.19	−0.43
Procheck G-factor (all dihedrals)^d^	−0.23	−1.36	−0.12	−0.71	−0.19	−1.12
MolProbity clashscore	16.28	−1.27	12.04	−0.54	13.26	−0.75

a Structural statistics were computed for ensembles of 20 deposited structures (PDB entries, SrfN: 2MA8, YahO: 2MA4, SssB-I: 2M2J2) using PSVS 1.4 [51], except as noted otherwise.

b Computed the expected number of typically observed resonance peaks, excluding: highly exchangeable protons (N-terminal, Lys, and Arg amino groups, hydroxyls of Ser, Thr, Tyr), carbonyl carbons of Asp, Glu, Asn, and Gln side chains, non-protonated aromatic carbons, and the C-terminal His_6_ tag.

c Average distance violations were calculated using the sum over *r* ^−6^.

d Ordered residue ranges [*S*(phi)+*S*(psi) >1.8] : SrfN:23–45,49–92; YahO: 23–45,49–73,84–90; SssB-I: 23–43,48–75,81–90.

e All (excluding tags) residue ranges: SrfN: 22–96; YahO: 22–91; SssB-I: 22–91.

MALDI-TOF mass spectrometry indicated that SrfN heterologously expressed in *E. coli* lacked the 21 N-terminal residues predicted to be part of the Sec signal peptide, apparently having been processed by cleavage of the peptide bond between residues 21–22, consistent with the prediction from the SignalP algorithm. Similar processing was found in 90% of YahO following heterologous expression in *E. coli*. Because they comprise a putative signal sequence for periplasmic localization, we presume the N-terminal 21 residues of these *Salmonella* proteins are recognized and cleaved by a homologous signal peptidase component of the *E. coli* machinery responsible for periplasmic protein export and maturation. Indeed, our top-down mode proteomics data show that the SrfN and YahO polypeptide sequences are similarly cleaved at the same positions during native expression *in vivo* in *Salmonella* [18]. Moreover, global proteomics studies using mass spectrometry to identify peptides from tryptic digestions of *Salmonella* also strongly indicated that the N-termini of SrfN and YahO were cleaved. These findings are consistent with the finding from a mass spectrometry-based proteomics analysis of potential *E. coli* biomarkers in food that the 21 N-terminal residues of *E. coli* YahO, which has 75% sequence identity to the *Salmonella* homologue, appear to be cleaved post-translationally [19]. The structures of SrfN and YahO were therefore refined without the N-terminal tail present, even though these residues are presumably present initially, immediately after expression.

The structure of SssB-I was determined with a construct expressing only residues 21–91 plus a short C-terminal His tag (LEHHHHHH), so there was never an N-terminal signal sequence to contend with. This confirms that for this protein at least, the N-terminal signal sequence is not required for proper folding of the DUF1471 domain.

### SssB-III structure determination by X-ray crystallography

In order to confirm that SssB contains three distinct DUF1471 domains, we subcloned the SssB fragments spanning residues 1–91, 108–175, and 244–314 corresponding to the N-terminal (SssB-I), central (SssB-II) and C-terminal (SssB-III) DUF1471 domains, respectively. The expression of all three SssB fragments resulted in production of soluble polypeptides of corresponding size by SDS-PAGE. Proper folding of these fragments was confirmed by ^1^H-^15^N HSQC experiments (data not shown) and the selenomethionine-enriched samples of these protein fragments as well as the full length SssB protein were submitted to crystallization. Protein crystals diffracting to resolution suitable for structure determination were only obtained in case of the fragment 244–314 corresponding to the SssB-III DUF1471 domain, and for this reason the solution state structure of SssB-I was solved by NMR spectroscopy as described above. Despite the fact that the crystals were pseudo-merohedrally twinned, the SssB-III structure was determined by single-wavelength anomalous diffraction. The final atomic model refined to 1.45 Å resolution and contained two molecules (A and B) in the asymmetric unit. The molecule A of SssB-III structure comprises residues Lys 247 to Glu 301 and Asn 305 to Lys 314 whereas molecule B comprises residues Lys 247 to Lys 314. In chain A, the region from Arg 302 to Asn 304 is not well defined in the electron density map and has not been modeled. In addition to the polypeptide chains, the asymmetric unit content includes 144 water molecules, two sulfate ions and one chloride ion. Details of the refinement are compiled in Table 2.

**Table 2:** X-ray data collection and refinement statistics for SssB-III.

Data collection	
Space group	*P*2_1_
Cell dimensions [Å],[°]	*a = *39.1
	*b = *52.5
	*c = *39.1
	β = 109.4
Temperature [K]	100
Radiation source	APS, ID-19
Wavelength [Å]	0.9793
Resolution [Å]^a^	50.0–1.45 (1.48–1.45)
Unique reflections	26,374 (1279)
R_merge_ b	0.079 (0.576)
<I >/<σI>	25.5 (2.2)
Completeness [%]	99.8 (100)
Redundancy	3.7 (3.6)
**Refinement**	
Resolution [Å]	36.92–1.45
Reflections work/test set	25,059/1,271
R_work_/R_free_ c	0.133/0.172
Twin fraction	0.204
No. of atoms protein/ligands/water	1116/11/144
Average B factor [Å^2^]	
protein/ligands/water	19.0/32.5/33.1
bond lengths [Å]	0.016
bond angles [°]	1.45
most favored	97.64
outliers	0
PDB entry	4EVU

a Values in parentheses correspond to the highest resolution shell.

b R_merge_ = Σ_h_Σ_j_|I_hj_–<I_h_>|/Σ_h_Σ_j_I_hj_, where I_hj_ is the intensity of observation j of reflection h.

c R = Σ_h_|F_o_|–|F_c_|/Σ_h_|F_o_| for all reflections, where F_o_ and F_c_ are observed and calculated structure factors, respectively. R_free_ is calculated analogously for the test reflections, randomly selected and excluded from the refinement.

### Common fold of DUF1471 proteins

**Figure 2:**
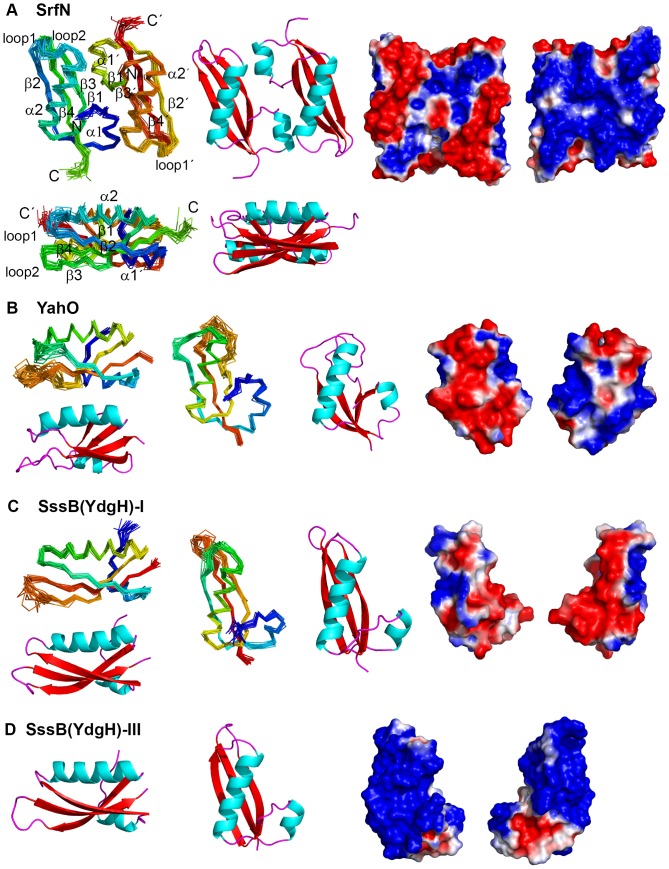
Structures and surface electrostatic characteristics of DUF1471 proteins. **A**: SrfN (dimer), **B**: YahO, **C**: SssB-I, **D**: SssB-III. In addition to ribbon cartoons colored by secondary structure, NMR structures show 20-member ensembles as Cα traces superimposed over the ordered residues determined by PSVS [51]. Electrostatic surface calculations were calculated with APBS using the PyMol plugin, with default parameters (0.15 M salt, kT/e = −1 to 1, pH 7.0) for the first members of the SrfN, SssB-I, and YahO ensembles and for SssB-III after conversion with PDB2PQR. The van der Waals surfaces are shown and are colored according to the charge (red for negative, blue for positive) on the water-accessible surface. Hydrogen atoms were added to SssB-III. The C-terminal 6x-His tags were removed and replaced with carboxylates for the calculation. N-termini for SrfN and YahO did not have the cleaved signal sequence, the N-terminus of SssB-III began at residue Lys 247.

All four experimentally-determined structures of DUF1471 domains adopt the same tertiary fold (Fig. 2) and can be superimposed on each other with 1.5–2.5 Å pairwise backbone rmsd over nearly all of the residues (Table 3). The fold features a three-stranded antiparallel β-sheet with another, shorter N-terminal β-strand parallel to strand 3, so that the strands are ordered 2-4-3-1 across the sheet, with strands 1 and 3 parallel. A short helix followed by a turn lies between strands 1 and 2. Strands 2 and 3 are linked by a long helix that lies atop the sheet, and strands 3 and 4 are linked by a reverse turn or loop (loop 2). Strand 3 contains a β-bulge at residues Ile 75 and Ala 76 (using numbering for residues in SrfN) that is also present at the equivalent position in both SssB-I and SssB-III, whereas YahO begins to adopts a less regular extended structure at this point leading into the following turn/loop (loop 2) following strand 3, which remains more disordered in the YahO structure than in the other NMR structures. In the SssB-III X-ray structure, residues in loop 2 and in the neighboring transition (loop 1) from strand 2 into the long helix exhibited particularly high B-factors and are likely to be locally flexible in solution. In SrfN on the other hand, there is little evidence in the NMR data for flexibility in this region, and the structure ensemble is converged in this loop. Loops 1 and 2 are found on the same end of the structure and together with the first, short helix correspond to the most variable regions of the sequence when DUF1471 paralogues are compared (Fig. 1). Notably, none of the residues conserved across DUF1471 paralogous subfamilies are in these two loops. Within most of the individual subfamilies themselves, there is high overall sequence similarity throughout, including in these loop regions. However, in a few of the subfamilies, including that of SrfN (Fig. 1), these loops are among the most variable regions in the sequence.

**Table 3:** Comparison of DUF1471 structures. Pairwise Dali [20] was used to align structures and calculate RMSD. The average NMR structures were used for purposes of structure comparison.

		SssB-I_ave	SssB-III	YahO_ave
**SrfN_ave**	rmsd	1.80	1.49	2.73
	#res aligned	66	63	68
	%id	21%	22%	26%
**SssB-I_ave**	rmsd		1.51	2.30
	#res aligned		68	63
	%id		28%	21%
**SssB-III**	rmsd			2.46
	#res aligned			70
	%id			26%

**Figure 3:**
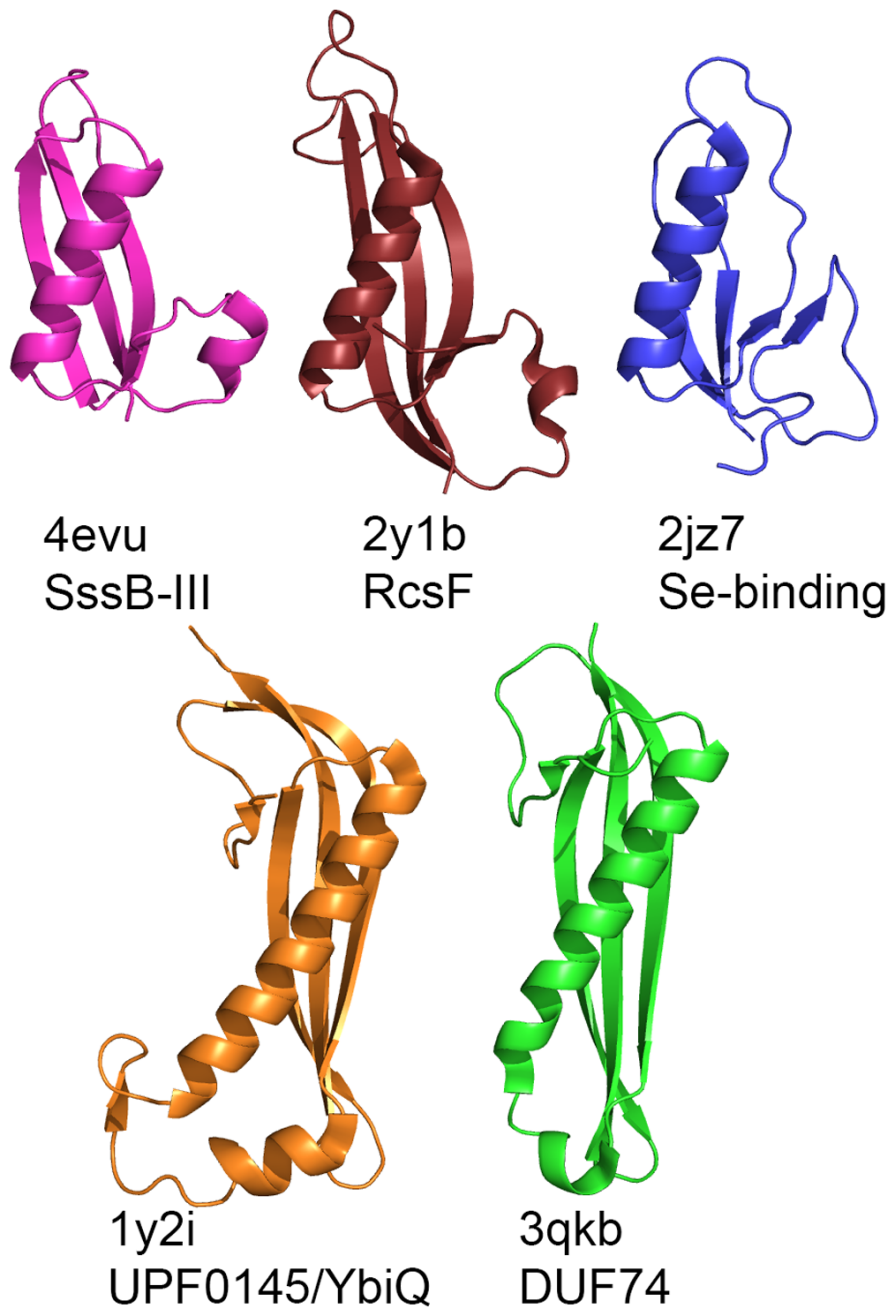
Proteins structurally similar to DUF1471 proteins. Similar structures were identified with Dali [20]. Additional details are presented in Table 4.

The fold adopted by DUF 1471 proteins is not novel, and several other proteins adopting this fold topology were identified using Dali [20] (Table 4). In particular, the structure of the bacterial outer membrane lipoprotein RcsF [21], [22], a regulatory system component containing redox-active disulfide bonds, aligns with the entire SrfN sequence with around 2 Å rmsd for backbone (N, Cα, C) atoms despite less than 10% sequence identity (Fig. 3). Cysteine residues are absent at the equivalent positions and are scarce overall in DUF1471 proteins. The only other notable difference is that RcsF has two longer loops at the end of strand 2 where the chain reverses direction and begins helix 2, and at the hairpin between strands 3 and 4. Both of these loops are adjacent to each other on the same end of the structure. Interestingly, RcsF clearly contains the same conserved β-bulge in strand three that is present in SrfN, SssB-I, and SssB-III. Three other classes of structurally characterized proteins were identified by Dali and determined by inspection to have the same fold as DUF1471 proteins (Table 4). They are a Se-binding protein from *Methanococcus vannielii* representing a small archaeal family [23], an uncharacterized bacterial domain (YbjQ/UPF0145) predicted to bind metals, and a bacterial domain of unknown function (DUF74). All three of these proteins appear to adopt pentameric structures. Two of them (YbjQ and the DUF74 domain) have more elongated β-sheets but lack the small first β-strand and alpha helix that is characteristic of DUF1471 and RcsF. Like the DUF1471 proteins and RcsF, both the DUF74 protein and the Se-binding protein, but not YbijQ, contain β-bulges in strand 3, though not at the equivalent positions in the strand.

**Table 4:** Protein structures from the Protein Data Bank that are similar (Z score >5.0) to DUF1471 proteins, determined with Dali [20]. Structures are X-ray structures except as noted.

protein	PDBentries	domain length(residues)	aligned length(residues)	oligomer	rmsd toSssB-III (Å)	Z-score
periplasmic domain of RcsF outer	2l8y (NMR)	86	71	monomer	2.2	6.3
membrane protein (*E. coli*)	2y1b	86	73		2.2	6.8
PFAM 11524 Archaeal Se-	2jz7	81	73	pentamer	2.8	5.8
binding protein						
UPF0145/YbjQ superfamily						
(*Bacillus cereus*)	1vr4	103	68	pentamer	2.7	6.1
	2gtc	103	69		2.7	6.1
(*Shigella flexneri*)	1y2i	107	69		2.4	5.3
DUF74 protein PCPN_1048	3qkb	92	67	pentamer	2.2	6.6
(*Pediococcus pentosaceus*)						

The structural similarity to RcsF is notable because of its suspected role in regulating capsule synthesis and association with other adjustments, such as colanic acid production and biofilm formation, due to changes in the extracellular environment [24], [25]. Similar processes have been found to be associated with DUF1471 proteins YcfR/BhsA, YbiM/McbA, YjfO/BsmA, and YhcN in *E. coli* and *Salmonella* [8], [10]–[16]. RcsF is distinctly different from DUF1471 proteins however in that it contains redox-active disulfide bonds. Furthermore, unlike SrfN it does not appear to be dimeric.

### Oligomeric state of SrfN, YahO, SssB-I and SssB-III

Several lines of evidence indicate that SrfN forms a dimer in solution throughout the pH range 5.0–7.4. Retention time in size exclusion chromatography and static light scattering recorded on the effluent from this column are consistent with a dimer. NMR measurement of the ratio of the ^15^N spin relaxation parameters T_1_ and T_2_ for estimation of the rotational correlation time τ_c_ [26] gave the following results that are consistent with a species of 17–18 kDa (the predicted dimer molecular mass, including the C-terminal His-tag is 18.2 kDa): at pH 5.0 and 25°C, 11 ns, and at pH 6.5 and 20°C, 12 ns. Samples of SrfN in pH 7.4 buffer yield ^1^H-^15^N HSQC spectra resembling those at pH 6.5, suggesting that the protein is still dimeric at the higher pH. Also, the dimeric form does not appear to be an artifact of the high protein concentration required for NMR spectroscopy: a 1 mM NMR sample diluted tenfold yielded a ^1^H-^15^N HSQC spectrum that was identical to the more concentrated sample. Furthermore, observation of intermolecular cross peaks in the 3-D ^13^C-edited ^13^C,^15^N-filtered [^1^H,^1^H] NOESY experiment suggests strongly that SrfN is dimeric in solution at pH values between 5.0 and 7.4. The SrfN dimer appears to dissociate and unfold at low pH, as the ^1^H-^15^N HSQC spectrum at pH 3.0 contains extra peaks suggesting a mixture of folded and unfolded forms is present (data not shown). It is not clear if the folded portion remaining at low pH 3.0 is monomeric or dimeric. Circular dichroism (CD) spectra collected at pH 7, pH 5, and pH 3 show diminished intensity at 222 nm at the lowest pH, suggesting greater random coil contribution. In the range of the 278 nm absorbance band from the Tyr side chain phenol, CD spectra were suggestive of a change in the environment surrounding one or more of the three Tyr residues in SrfN, two of which are near the dimer interface.

In contrast to SrfN, YahO and SssB-I are clearly monomeric in the conditions under which the structures were determined. Rotational correlation time τ_c_ = 6 ns was calculated from ^15^N spin relaxation parameters T_1_ and T_2_ [26] measured at 25°C for YahO, SssB-I and Sssb-III, which is consistent with a monomeric protein. Retention times in size exclusion chromatography for YahO, SssB-I, SssB-III and full-length SssB also were consistent with a monomeric species. The SssB C-terminal domain (SssB-III) adopts a dimeric form in the X-ray structure. However, the relative arrangement of monomers and the dimer interface are different from those of SrfN (Fig. S1), and likely represent a crystallization artifact.

While the four structures determined here have a similar domain fold, the dimeric nature of SrfN contrasts with the monomeric YahO, SssB-I, and SssB-III structures found in our studies. The dimer interface in SrfN is not particularly rich with hydrophobic residues, and there are several buried polar residues that appear to be involved in a network of intra- and inter-molecular hydrogen bonds and ion pairs. Such an interface could conceivably be disrupted by high salt concentrations. Indeed, a sample of ^15^N-labeled SrfN in pH 5.5 ammonium acetate buffer with 0.5 M NaCl yielded an estimated τ_c_ (at 25°C) of 9 ns, approximately midway between the values measured for dimeric SrfN in low salt and monomeric YahO and SssB-III, indicating that the dimeric species may be partially disrupted. The existence of different oligomerization states among paralogous proteins is not uncommon, and dimerization in SrfN may be a property enabled by the fold as the need arose during sequence divergence following gene duplication and subsequent adaptation and evolution of new physiological roles. Consistent with this, the sequence alignment of the DUF1471 sequences from different subfamilies shows that none of the SrfN residues at the interface are conserved. However, the crystallographic dimer found for SssB-III (Fig. S1) has an interface indicative of an auxiliary role in complex formation (the complex formation significance score calculated in PISA is 0.206) that may suggest a propensity for opportunistic dimer formation, depending on the conditions. Finally, because the N-terminus of SrfN (Ala 22) does not protrude from the surface, but is actually somewhat buried and would likely be inaccessible to a protease, it follows that *in vivo* dimerization must occur after cleavage of the N-terminal signal peptide.

### Surface characteristics of SrfN, YahO, SssB-I, and SssB-III and interactions with sulfated polysaccharides

Rudd and coworkers [1] identified what is now the DUF1471 family quite early in the microbial genome era, probably because the *E. coli* genome had numerous paralogues that could be clustered. They identified several sequence motifs that appear to characterize the entire family, but mapping these onto the structures reported herein does not clearly reveal significant clustering of conserved residues on the surface across multiple subfamilies. Overall, only a handful of residues are substantially conserved throughout the superfamily (Fig. 1). Most appear to be interior hydrophobic residues probably associated with maintaining stability of the fold. The loops are notably diverse from a sequence standpoint, and vary in length in different subfamilies. Within subfamilies however, sequence similarity is characteristically high overall, including residues in some loop regions and on the surface (Fig. S2). Therefore, surface characteristics common to all members of a family can be inferred from the structures determined here. These conserved features may reflect one or more substrates or interacting partners that are common to members of the subfamily.

**Figure 4:**
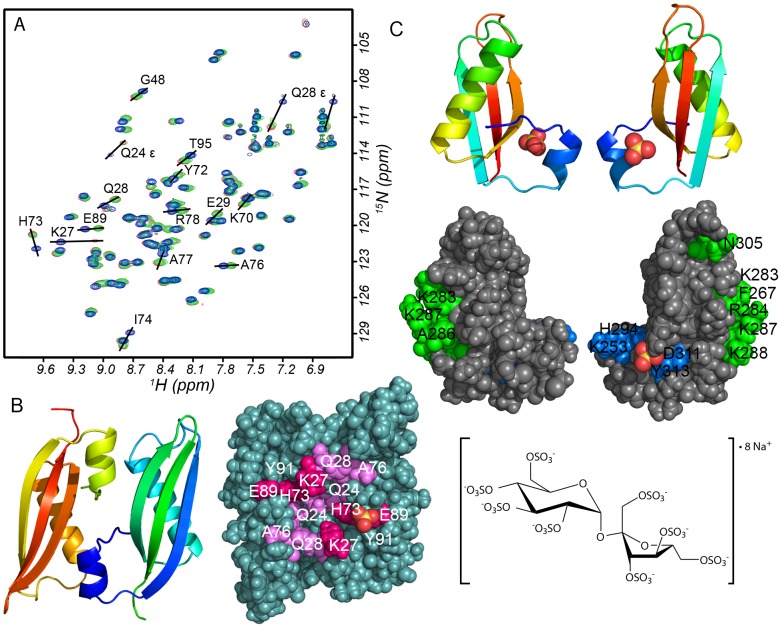
Addition of ligands to SrfN. **A**: SrfN–sucrose octasulfate titration monitored with 2-D ^1^H-^15^N HSQC. Superimposed spectra: blue, SrfN only; green and red, SrfN +5x and 10x molar excess sucrose octasulfate. **B**: Chemical shift perturbations following sucrose octasulfate (shown at right) addition mapped onto SrfN surface; the perspective is the same as in Fig. 2 where the positively-charged surface is shown (blue). Ribbon cartoon of SrfN from the same perspective is shown adjacent to the surface depiction. Side chains colored violet have >1 linewidth shift with sucrose octasulfate and similar shifts with heparin & high [SO_4_^2−^] but are not conserved in SssB-III (Q24, Q28, A76). Side chains colored magenta have >1 linewidth shift and are conserved in SssB-III (K27/253, H73/294, E89/D311). A fourth conserved residue at the SO_4_-binding position (Y91/313) from the SssB crystal structure does not show chemical shift perturbation upon sucrose octasulfate addition to SrfN. The sulfate ion was positioned by a superposition of the SssB-III crystal structure on SrfN. **C**: Chemical shift perturbations in SssB-III upon titration of sucrose octasulfate, showing that interactions occur not at the sulfate-binding site common to SrfN, but at a patch of basic residues some distance away.

The electrostatic surface properties of the SrfN structure at neutral pH reveal a region of positively charged residues that almost completely covers one face of the protein, while the opposite face has two ridges of negative charge formed by the two long helices on that side. The region of positive charge appears to surround a small pocket at the dimer interface and on the dyad symmetry axis that is surrounded by basic residues that could be interpreted as a potential binding site for a small negatively charged ligand (Figs. 2 and 4). The residues forming this pocket are conserved (Fig. 1, panel C). YahO (pI 7.9), though monomeric, has similarly separated positive and negative surfaces on the equivalent sides of the protein, while SssB-I (pI 5.2) has a somewhat different distribution of positively and negatively charged regions and the SssB-III (pI 9.3) surface is positively charged everywhere except on one end of the protein near the C-terminus consisting of residues in the first helix and the linking segment before the second β-strand, which is negatively charged. The equivalent regions in SrfN, YahO, and SssB-I also are negatively charged (Fig. 2). Homology models of other DUF1471 proteins indicate the diversity in surface electrostatic characteristics throughout the family (Fig. S3), suggesting their intermolecular associations are likely to be diverse as well.

There was no evidence, such as unassigned resonance peaks in NMR spectra or extra volume in the (X-ray) electron density map, for bound ligands that copurified with SrfN, YahO, SssB-I, or SssB-III. However, because several studies of other DUF1471 homologues suggested links to biofilm formation and other aspects of the extracellular matrix [8], [14], [15], and in light of the evidence that both SrfN and SssB are secreted by *Salmonella* in association with pathogenesis [2], [5], we considered whether a common function, broadly speaking, could exist in direct interactions with extracellular polysaccharides. After all, both bacterial biofilms and extracellular matrices in eukaryotic hosts are characteristically rich with diverse types of polysaccharides. Several DUF1471 proteins are substantially up-regulated in biofilm-forming conditions, suggesting that the high quantities of protein are required, perhaps for interaction with an abundant substrate. Moreover, the diverse assortments of paralogous subfamilies within DUF1471 that appear in different genera in the Enterobacteriaceae could point towards a similarly diverse set of substrates, such as the numerous distinct extracellular polysaccharides encountered by these bacteria in the various environments they populate.

Because one face of SrfN was so distinctly basic and appeared to have a small positively charged pocket in the middle (Fig. 2), we hypothesized that it could interact with anionic polysaccharide ligands such as glycosaminoglycans. We screened for SrfN binding to the polysaccharides mucin, alginic acid, hyaluronic acid, chondroitin sulfate, and heparin sulfate. We also screened against compound libraries of the twenty common amino acids, as well as common monosaccharides and disaccharides. Ligand interactions were tested for by monitoring resonance peak broadening in 1-D ^1^H spectra of ligand mixtures upon addition of SrfN. Among the aforementioned additives, we found evidence of interaction only with heparin sulfate. Subsequently, ^1^H-^15^N-HSQC spectra were monitored upon titration of heparin as well as heparin disaccharide and hexasaccharide, sucrose octasulfate, and sodium sulfate. Sucrose octasulfate is commonly used as an inexpensive heparin mimetic and is believed to bind similar sites in heparin-binding proteins such as fibroblast growth factor [27]. Chemical shift perturbations of a subset of the peaks in the HSQC spectra upon addition of the smaller ligands suggested specific interactions with SrfN. Similar NMR chemical shift perturbations have been used previously to characterize specific interactions of known heparin binding proteins with sulfated heparin oligosaccharides or sucrose octasulfate [28]. These experiments indicate qualitatively that there is a region on the surface which interacts only weakly with sulfate ion at neutral pH, because a large (>200-fold) excess of SO_4_^2−^ caused only small chemical shift perturbations, while small excesses (around five-fold) of sucrose octasulfate, heparin hexasaccharide, and heparin (based on the calculated concentration of hexasaccharide units) caused substantial perturbations of the same residues’ amide shifts. Induced chemical shifts were nearly maximal at sucrose octasulfate concentrations of around 1 mM when SrfN (dimer) was 0.25 mM (Fig. 4, panel A and Fig. S4 panel B), so the K_d_ of this complex can be roughly constrained to a value substantially less than 0.5 mM. A similar pattern of amide chemical shift perturbations was observed incidentally upon buffer exchange from phosphate to Tris buffer, indicating the same oxyanion binding site on each SrfN monomer. The interaction with heparin disaccharide appears to be somewhat weaker, as greater excesses caused comparatively minor chemical shift perturbations relative to those of sucrose octasulfate, heparin hexasaccharide, and heparin. However, because there are multiple forms of heparin disaccharide, reflecting different possible combinations of sulfate ester modifications in heparin as well as the chemical structure of the reducing and non-reducing ends, which would be normally linked to adjacent residues in polymeric heparin, it is possible that the disaccharide used is not the preferred form. Alternatively, the optimal interaction motif could be larger than two monosaccharide residues. Interaction with heparin sulfate polysaccharide showed not only the same chemical shift perturbations seen with the smaller fragments, but also showed substantial line broadening in resonance peaks in the ^1^H-^15^N HSQC (Fig. S4), with the exception of side chain amide peaks and a few backbone amides near the C-terminus, indicating slower tumbling of the protein due to interaction with the polysaccharide. (The estimated average molecular weight of the heparin sulfate is 15–30 kDa.) Notably, SrfN did not show evidence of interaction with chondroitin sulfate, another sulfated polysaccharide. Even at 10 mg/mL chondroitin sulfate, corresponding to 8 mM concentration of the repeating hexasaccharide unit (around 10–20-fold excess), no line broadening was apparent and the estimated correlation time was 14 ns, slightly larger than SrfN alone and probably attributable to bulk solvent effects from the high concentration of the polymer, rather than to specific binding. SrfN was also found to bind to immobilized heparin (GE HiTrap Heparin-HP column) with considerable affinity, requiring 300 mM NaCl for elution at pH 7, and 1.5 M NaCl at pH 5. These results are seen as a propensity for interaction with polymeric ligands bearing multiple negatively charged groups, and may point towards a specific interaction with one of the various motifs present in heparin- or heparan-like glycosaminoglycans or proteoglycans that are common in eukaryotic host environments encountered by enteropathogenic bacteria. We also screened for binding to proteins from a host-cell lysate using mass spectrometry to identify host proteins that were pulled down from the lysate, but found no significant positive hits (R.N. Brown, personal communication).

Superposition of the SrfN and SssB-III structures revealed that SrfN residues perturbed by sucrose octasulfate and heparin-hexasaccharide binding coincided with SssB-III residues surrounding one of two bound sulfate ions in the crystallographic dimer. (The crystallization buffer contained 1.9 M ammonium sulfate). Four of these residues were conserved between the two proteins (Fig. 4), despite low overall sequence similarity between the two proteins. Titration of sucrose octasulfate into a ^13^C,^15^N-labeled sample of SssB-III showed amide ^1^H and ^15^N chemical shift perturbations consistent with a localized interaction with the protein, similar to what was found with SrfN. However there was no evidence from estimation of the correlation time that the interaction induced dimerization. Furthermore, mapping the perturbed residues onto the structure suggested the interaction was with a zone of basic residues located around the side of the protein (Fig. 4, panel C) that is distinct from the sulfate binding site found in the crystal structure. Interestingly, SrfN has several of these residues in common with SssB-III. We suggest that because SssB is not a dimer, sucrose octasulfate cannot bridge the two sulfate binding sites as it can in SrfN, and it binds more favorably to the patch of basic residues found. In SrfN, these residues may be involved in binding, for example, longer heparin chains that can span not only both sulfate sites but also the two basic patches on either side. Though heparin and sucrose octasulfate are so highly negatively charged that any protein with some patch of basic residues probably interacts with them to some degree, the affinity for heparin and the magnitude of the secondary chemical shifts upon heparin binding that are seen with SrfN resemble what is found for known heparin binding proteins, particularly at pH 5.0 where the His residues forming the anionic pocket (Fig. 4, panel B) would likely be protonated.

### Modeling of the SrfN and SssB-III interactions with ligands

**Figure 5:**
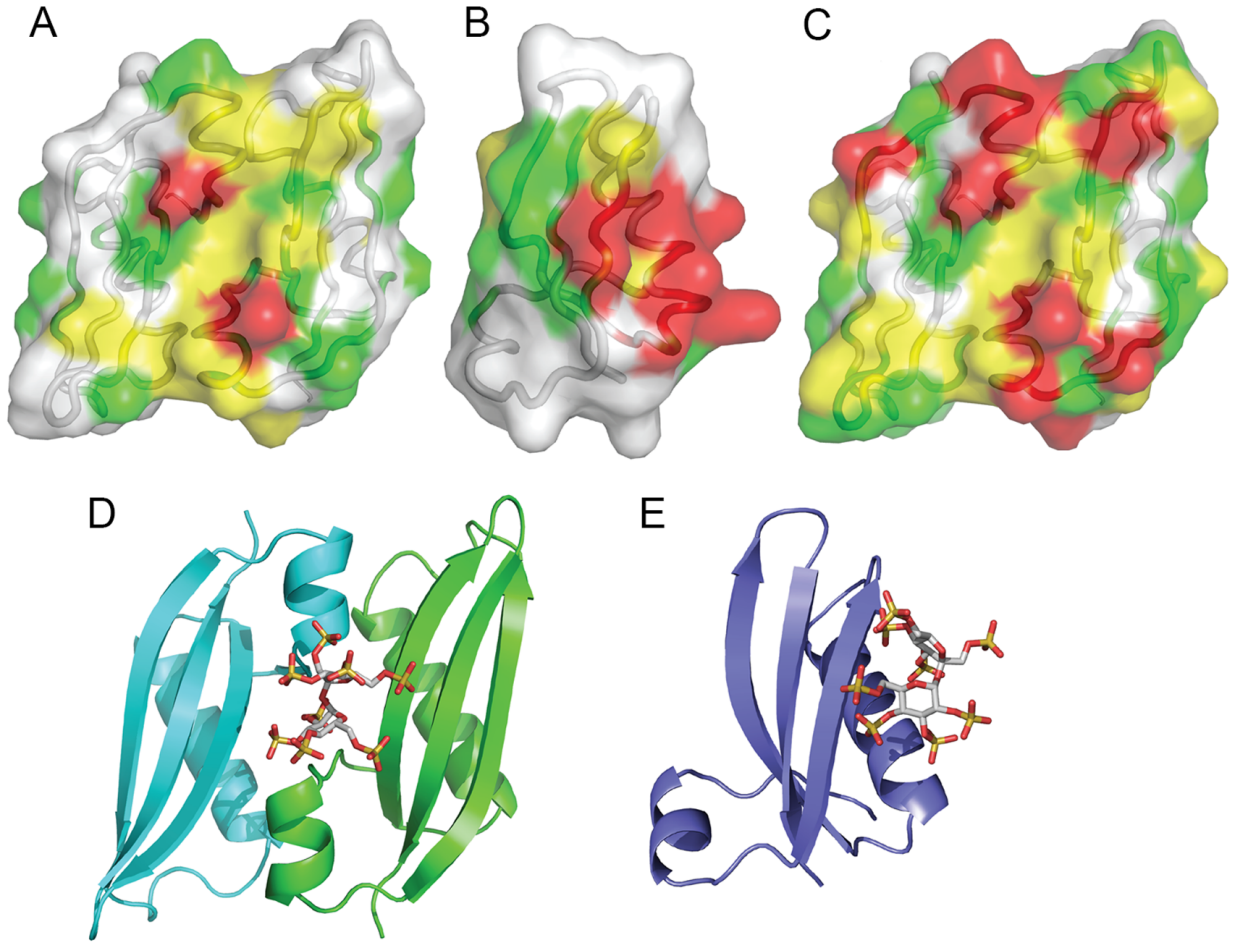
Ligand binding sites predicted by coarse-grained simulations. Predicted sucrose octasulfate interactions with (**A**) SrfN and with (**B**) SssB-III, and (**C**) predicted maltohexaose dodecasulfate interactions with SrfN. Red, yellow, and green indicate binding score levels of >0.1, >0.05, and >0.02, respectively. **D** and **E**: Low energy structures of models shown in panels A and B after being reverse-mapped to atomistic sucrose octasulfate in the space occupied by the coarse-grained equivalent, followed by 1000 steps of vacuum minimization in GROMACS [73] using the CHARMM force field to eliminate clashes, with ligand parameters were derived using SwissParam [74]. The all-atom models are shown for illustrative purposes and do not necessarily indicate global free energy minima at all-atom resolution.

While the isoelectric point of mature SrfN is slightly acidic (pI 6.3), SssB-III (pI 9.5) has a high net positive charge at neutral pH and might be expected to interact nonspecifically with polyanionic ligands of any kind. To better understand the observed differences, the interactions of SrfN and SssB-III with small sulfated polysaccharides were modeled using a coarse-grained approach without any constraint from the chemical shift perturbations that had been observed. This modeling identified the same binding sites in each protein inferred from experimental data, supporting the findings suggested by NMR chemical shift perturbations that by providing two binding sites for sulfate esters, the SrfN dimer binds sucrose octasulfate and longer heparin polysaccharides in a way that spans the center of the dimer interface on the positively charged face of the protein (Fig. 5, panels A, C, and D), whereas monomeric SssB-III does not provide the cooperativity afforded by having two sulfate binding sites on either side of a pliable dimer interface, and instead interacts most strongly with the ligand at the distal Lys-rich site (Fig. 5, panels B and E). The modeling also appears to support the apparent higher affinity of longer heparin polysaccharides for SrfN relative to heparin disaccharide. The hexasaccharide binding site includes that for the sucrose octasulfate disaccharide, but the high-affinity residues span a much larger area across the basic side of SrfN, possibly in two symmetry-related modes; these characteristics may enable a larger binding area and a higher binding affinity. (Fig. 5, panel E).

## Conclusion

In *E. coli*, the DUF1471 proteins YcfR/BhsA, YbiM/McbA, YjfO/BsmA, and YhcN appear to be involved in the bacterial response to extracellular stress. They confer various forms of resistance compared to mutants where the corresponding genes are inactivated [8], [12]–[15], and several are up-regulated in *E. coli* as well as *Salmonella* following exposure to oxidative and other stresses [9]–[12]. The mechanisms by which DUF1471proteins function in conjunction with these phenomena are unclear, but in general they seem to either directly or indirectly influence and change characteristics of the cell surface. For example, attenuation of biofilm formation together with increased production of colanic acid and concomitant mucoidy was associated with YbiM/McbA activity in *E. coli* [14]. Conversely, YcfR/BhsA appears to influence surface characteristics that mediate surface attachment and cell aggregation [12], behaviors that typically precede biofilm formation.

We may speculate that SfrN and SssB expression are similarly influenced by stress and thereby mediate response and resistance to stress in *Salmonella*, perhaps in response to particular stresses experienced during invasion of host cells. Both SrfN and full-length SssB modestly enhance *Salmonella* virulence relative to their individual deletion mutants in mouse infection models. Such enhancement could result from modulation of the host response by interaction with host proteins, as seen for many pathogen secreted effectors, or by protecting the bacterial cell from external stresses, as seen in several other DUF1471 proteins. We suggest that establishment of intracellular infection and protection from the host cell response rely on parallel aspects of the same mechanisms that control biofilm formation, given the involvement of several *E. coli* DUF1471 paralogues in such processes apart from pathogenesis as detailed above. Furthermore, because SrfN has small number of close homologues, unlike other DUF1471 proteins, we propose that SrfN in particular represents an adaptation enabling host colonization, probably as the result of a gene duplication event that occurred after divergence of the *Escherichia* lineage from either *Salmonella* or some other genus from which SrfN was later acquired by *Salmonella* via horizontal transfer.

YahO appears not to be specifically involved with pathogenesis and therefore is likely to be functionally more closely related to YcfR/BhsA, YbiM/McbA, YjfO/BsmA, or YcfR with an uncharacterized role in general or specific stress response and biofilm formation. On the other hand, SrfN and SssB do appear to be associated with pathogenesis, and SrfN has been shown here to interact particularly strongly with heparin and the heparin surrogate sucrose octasulfate. This observation may point towards biochemical function, insofar as heparin is a major component of the epithelial extracellular matrix, an environment through which *Salmonella* must navigate during host colonization and establishment of infection. Furthermore, this could imply a role for SrfN in recognition of the host cell–a prerequisite for activation of Type III secretion systems during pathogenesis. The structures and chemical shifts reported here will be important tools for developing an understanding of the role of SrfN and other DUF1471 proteins as their functions and interactions with other biomolecules are discovered.

## Materials and Methods

Stable isotope-labeled compounds were obtained from Cambridge Isotope Laboratories (Andover, MA). Sucrose octasulfate and heparin hexasaccharide were obtained from Carbosynth (Compton, UK). Heparin from porcine intestine, heparin disaccharide I-S disodium salt (α-ΔUA-2S-[1→4]-GlcNS-6S), chondroitin sulfate, and other biochemicals were obtained from Sigma (St. Louis, MO).

Stable isotope-labeled samples of SrfN and YahO for NMR structure determination were prepared in a manner similar to that which has described previously [29]. Briefly, the coding sequences of the STM0082 (SrfN) and SC0077 (YahO) genes of *Salmonella enterica* serovars Typhimurium LT2 and Choleraesuis, respectively, were cloned into vector pET21_NESG coding for a C-terminal affinity tag (LEHHHHHH) to yield the plasmids StR109-21.1 and StR106-21.1. These expression plasmids have been deposited in the PSI Materials Repository (http://psimr.asu.edu/). The sequence of YahO from *Salmonella* Typhimurium LT2 (STM0366) is identical to SC0077. The plasmids were transformed separately into *E. coli* BL21(DE3) pMgK cells and cultured in MJ9 medium [30] containing 2 g/L [*U*-^13^C]-glucose and 1 g/L [*U*-^15^N]-NH_4_Cl. Biosynthetically-directed ^13^C-labeling was accomplished with minimal media containing 5% *U*-^13^C glucose and 95% unlabeled (natural abundance ^13^C) glucose [31]. Cells were grown initially at 37°C in 1 L culture volume, and protein expression was induced at 17°C by 1 mM isopropyl-β-D-thiogalactopyranoside (IPTG) at mid-log phase growth. Protein was purified using Ni^2+^ affinity chromatography (Ni-NTA) followed by size exclusion chromatography. Yields after purification were 50–95 mg/L for SrfN and 10–20 mg/L for YahO. SrfN NMR samples were prepared in pH 6.5 20 mM MES with 100 mM NaCl and 5 mM CaCl_2._ YahO NMR samples (ca. 1 mM concentration) were prepared in 20 mM ammonium acetate pH 4.5, 100 mM NaCl, 5 mM CaCl_2_, 10% D_2_O.

To prepare samples of SrfN for characterization by mass spectrometry, confirmation and refinement of the original NMR structure, and characterization of binding interactions, the recombinant full length STM0082 (SrfN) gene was synthesized by GenScript (Piscataway, NJ) and incorporated into the pGS-21a expression vector encoding a C-terminal 6X-His tag. The plasmid was transformed into *E. coli* BL21 Star (DE3) cells (Invitrogen, Carlsbad CA). Unlabeled protein was expressed by autoinduction [32] in 1 L cultures of ZYP-5052 media (ZY stock, 1 mM MgSO_4_, 1X metals mix, 1X 5052, 1X NPS, 50 ug/mL ampicillin) grown at 37°C with shaking. [*U*-^15^N,^13^C]-SrfN was produced by expression in M9 minimal media containing 2 g/L [*U*-^13^C]-glucose and 1 g/L [*U*-^15^N]-NH_4_Cl and supplemented with 10 µM Fe^3+^ and 7 µM Zn^2+^. Cell pellets were resuspended in lysis buffer (20 mM sodium phosphate, 500 mM NaCl, 5 mM imidazole, pH 7.4) plus Halt protease inhibitor cocktail (Thermo Fisher, Waltham MA). Cells were lysed by passage three times through a French press at 8,000 PSI and the cell-free extract was applied to a 5 mL HisTrap HP column (GE Healthcare, Little Chalfont UK). Bound protein was washed with 75 mL of lysis buffer and eluted with elution buffer (20 mM sodium phosphate, 500 mM NaCl, 500 mM imidazole, pH 7.4). SrfN-containing fractions were diluted with buffer (20 mM sodium phosphate, 100 mM NaCl, pH 6.5) and further purified by cation exchange on a 5 mL HiTrap SP HP column (GE Healthcare). Purity of the SrfN preparation was analyzed using SDS-PAGE and mass spectrometry and SrfN was exchanged into one of several buffers by centrifugal ultrafiltration. Buffers included PBS (pH 7.4), pH 5.0 20 mM NH_4_OAc with 100 mM NaCl, and pH 6.5 20 mM MES with 100 mM NaCl and 5 mM CaCl_2_. Structural studies were conducted in the pH 6.5 buffer. Samples were concentrated to 0.5–1.0 mM for NMR spectroscopy.

Analytical size exclusion chromatography and static light scattering were used to assess oligomeric state of SrfN and YahO as described [33], using a Shodex KW-802.5 column with 100 mM Tris pH 7.5 buffer containing 100 mM NaCl. Thirty µL of concentrated protein (8–12 mg/mL) were used for analysis. Titrations of heparin, heparin hexasaccharide, heparin disaccharide, sucrose octasulfate, and sodium sulfate were accomplished by making concentrated stocks of the ligands in NMR buffer and adding small aliquots to NMR samples. Binding of SrfN to heparin was assayed using a 1 mL HiTrap heparin column (GE Healthcare) with a linear NaCl gradient from 150 mM to 2 M. Sodium-EDTA was added to a 0.2 mM [*U*-^13^C,^15^N]-SrfN sample to assess whether a bound metal influenced the structure.

The gene fragments coding for residues 21–91 and 244–314 of SssB were cloned from genomic DNA from *S.* Typhimurium strain LT2 according to the standard protocols developed at the Midwest Center for Structural Genomics (MCSG), as described previously [34]. The expression plasmids for SssB domain I (SssB-I) and SssB domain III (SssB-III) polypeptides were cloned into pET21b and transformed into *E. coli* BL21-Gold (DE3) (Stratagene, La Jolla CA), which harbors an extra plasmid (pMgK) encoding three rare tRNAs (AGG and AGA for Arg, ATA for Ile). The *E. coli* cells expressing SssB-I and SssB-III for NMR samples were then cultured in 1 L minimal media containing [*U*-^13^C]-glucose and ^15^NH_4_Cl as described above for SrfN and YahO, while those expressing SssB-III for crystallization trials were cultured in 1 L LB growth medium supplemented with ampicillin (100 µg/mL) and kanamycin (50 µg/mL), and incubated at 37°C with shaking until the culture reached an OD_600_ of 0.6–0.8. At this point the culture was induced with 0.4 mM IPTG and allowed to grow overnight at 15°C. Cells were harvested by centrifugation, disrupted by sonication, and the insoluble material was removed by centrifugation. Selenomethionine-enriched SssB-III was prepared similarly after growth of bacteria in SeMet high-yield media (Shanghai Medicilon, Shanghai China). The SssB-I and SssB-III fragments were purified using Ni-NTA affinity chromatography. [*U*-^13^C,^15^N]-SssB-I and [*U*-^13^C,^15^N]-SssB-III were exchanged into NMR buffer (10 mM pH 6.5 Bis-Tris buffer containing 200 mM NaCl, 10 µM ZnSO_4_, 10 mM DTT, 1 mM benzamidine, and 7% D_2_O). Unlabeled SssB-III for crystallization trials was dialyzed and stored in a buffer containing 10 mM HEPES (pH 7.5), 500 mM NaCl and 0.5 mM TCEP.

The structures of SrfN, YahO, and SssB-I were determined by solution state NMR spectroscopy, while that of SssB-III was determined by X-ray crystallography. However, NMR data sufficient to make backbone and side chain chemical shift assignments was collected for SssB-III for use in probing ligand interactions.

NMR spectra of SrfN, YahO, and SssB-III were acquired at 20 or 25°C on Varian Inova 600 and 750 instruments as described previously [35], [36]. Rotational correlation times used to characterize the oligomerization state of SrfN, YahO, and SssB-I were measured using ^1^H-detected 1-D ^15^N relaxation experiments [26]. The effect of protein concentration on the oligomerization state of SrfN was assessed by comparing the ^1^H-^15^N HSQC spectrum of a sample of approximately 1 mM concentration to that of a sample of tenfold lower concentration. Data was processed with PROSA [37], NMRPipe [38] and Felix (FelixNMR, San Diego CA). Sequence-specific resonance assignment of SrfN and YahO was performed in a semi-automated fashion using AutoAssign [39] and CARA [40]. Structures of SrfN and YahO were calculated using automatic NOE assignment in AS-DP [41] and CYANA [42], followed by iterative manual refinement using CYANA, and final refinement with Xplor-NIH [43] and CNS [44], [45] as previously described [35], [36].

NMR spectra of SssB-I were collected at 25°C on an 800 MHz Bruker Avance spectrometer equipped with a cryogenic probe. Three-dimensional spectra were acquired using non-uniform sampling in the indirect dimensions and reconstructed with the multi-dimensional decomposition software MddNMR [46], [47], interfaced with NMRPipe. Backbone assignments were determined using the program FAWN [48] from HNCO, CBCA(CO)NH, HBHA(CO)NH, HNCA and ^15^N-edited NOESY-HSQC spectra. Aliphatic and aromatic side chain assignments were completed with the aid of (H)CCH-/H(C)CCH-TOCSY and ^13^C-edited NOESY spectra, respectively. Structure calculations were initially performed using the program CYANA 3.0 integrated with the noeassign module for automated NOE assignments [42]. Backbone torsion angle restraints were derived from chemical shifts using the program TALOS+ [49]. Distance restraints were obtained from cross-peaks in ^15^N- and ^13^C-edited NOESY spectra. The best 20 of 100 CYANA structures from the final cycle were subjected to restrained molecular dynamics simulation in explicit water by the program CNS [44], [45]. Quality of the final structures of SrfN, YahO, and SssB-I was assessed with RPF scores [50] and PSVS [51]. Statistics for these structures are reported in Table 1.

The structure of SssB-III was determined by X-ray crystallography by the Midwest Center for Structural Genomics [52]. Crystals of SssB-III were grown by vapor diffusion in hanging drops at room temperature. The reservoir buffer (0.1 M sodium citrate pH 5.6, 0.2 M K/Na tartrate, 1.9 M (NH_4_)_2_SO_4_, 5% glycerol, and trypsin protease (7.5 µg/mL)) was mixed with an equal volume of protein solution (9 mg/mL). Crystals were placed in a cryoprotectant solution (Paratone-N oil) and then cooled in a stream of cold nitrogen vapor. The X-ray diffraction experiments were performed at the Structural Biology Center ID-19 beamline at the Advanced Photon Source, Argonne National Laboratory. The single-wavelength anomalous diffraction (SAD) dataset was collected at 100K at the selenium K-absorption edge to 1.45 Å resolution. The diffraction data were integrated and scaled in the HKL-3000 suite [53]. Intensities were converted to structure factor amplitudes in the Truncate program from the CCP4 package [54]. The R_free_ set was selected in thin resolution shells by an appropriate procedure implemented in the Phenix package [55]. The processing statistics are given in Table 2. The structure was solved by the SAD method using selenium peak data and the HKL-3000 software pipeline [53]. Selenium sites (three per protein monomer) were localized by SHELXD and the handedness was determined SHELXE [56]. Phasing was performed in MLPHARE [57] and was followed by density modification procedure in DM [58]. The initial protein model was built in ARP/wARP [59]. Manual model rebuilding was carried out in Coot [60] while maximum likelihood crystallographic refinement with anisotropic B factors for all atoms was done in Refmac5 [61]. The occupancies of residues in multiple conformations were refined in Phenix.refine [55]. As the data revealed pseudo-merohedral twinning with a twin law (*l*, *−k*, *h*), the refinement protocol included twin option. The final twinning fraction is *α* = 0.204. The minimization converged with *R*-factor of 0.133 (*R*_free_ = 0.172). The quality of the final model was verified using the MolProbity server [62]. The refinement statistics are given in Table 2.

Protein mass spectrometry was conducted as described previously [18]. Circular dichroism spectra were collected in 1 mm cells on an Aviv (Lakewood, NJ) Model 410 spectropolarimeter.

Sequence searches with BLAST and PSI-BLAST typically used only the mature form of the protein–the putative N-terminal signal peptide sequence was not included in the query. Prediction of signal peptide sequences was accomplished with SignalP [63]. The DUF1471 family phylogenetic tree was constructed with T-REX [64] using the neighbor joining method [65]. Surface electrostatic features were analyzed with APBS [66] and PDB2PQR [67] and visualized with PyMol [68]. Dali [20] was used for structure similarity comparisons. Dimer interfaces were analyzed with PISA [69].

SrfN and SssB-III interactions with ligands were modeled with the MARTINI 2.1 force field [70]. The experimentally determined structures were converted to MARTINI coarse-grained representations using the martinize.py script, and near-native structure was maintained by using elastic bonds between C_α_ atoms less than 0.9 nm apart with a force constant of 500 kJ/mol/nm^2^, as described previously [71]. Coarse-grained models of sucrose octasulfate and maltohexaose dodecasulfate, intended to mimic heparin hexasaccharide, were derived from sucrose and maltoheptose, respectively, in the MARTINI 2.0 force field for carbohydrates [72]. To mimic carbohydrate sulfation, (Qa) groups were attached to each available hydroxyl position (two per coarse-grained monosaccharide residue) with a bond length of 0.47 nm and a cosine-squared bond angle term centered about 140° with a force constant of 25. For each of 50 independent starting configurations, the ligand was randomly positioned and oriented with a 1.5 nm solvent layer separating the outer edges of protein and ligand. MARTINI water and sufficient ions were added to neutralize the system. Each simulation was carried out for 500 ns using a time step of 20 fs with GROMACS [73]. For analyses, the last 400 ns from each simulation were aggregated, and the number of frames for which each residue contacts at least one atom of the ligand was counted. The binding score of each site was then quantified as the fraction of all trajectory frames for which that residue contacts the ligand. Results of coarse-grained simulations were converted back to all-atom models for easier visualization.

## Supporting Information

Figure S1 **Comparison of solution-state SrfN dimer with crystallographic dimer for SssB-III.** Ribbon cartoon depictions of SrfN dimer (left) and SssB crystallographic dimer (right).
(PDF)

Figure S2 **Strictly conserved residues in the SrfN subfamily.** Residues conserved in all sequences shown in Fig. 1, panel C are mapped onto the structure as light blue spheres.
(PDF)

Figure S3 **Homology models of other DUF1471 proteins from** ***Salmonella*** **and YbiM from** ***E. coli*** **.** The models indicate the diversity of surface electrostatic characteristics across the family. The surfaces were calculated and are displayed in the same way described in the Fig. 2 caption.
(PDF)

Figure S4 **^1^H-^15^N HSQC spectra of SrfN titrations with ligands.** SrfN monomer concentrations are indicated, while the final ligand:SrfN dimer molar ratios are given in parentheses. **A**: 0.5 mM SfrN (blue) titration with 100 mM (green, 400∶1) and 200 mM (red, 800∶1) Na_2_SO_4_. **B**: 0.5 mM SrfN (blue) w/5 mM heparin disaccharide (green, 20∶1) and 4 mM hexasaccharide (red, 16∶1). **C**: 0.5 mM SrfN (blue) titration with 0.4 mg/mL heparin polysaccharide (green, 0.6 mM heparin disaccharide equivalent, approximately 2∶1 ligand/SrfN dimer ratio) and 2.1 mg/mL heparin polysaccharide (red, 3.1 mM disaccharide equivalent, approximate ratio 12∶1). **D**: expanded portion of spectrum highlighted in panel C. **E**: 0.2 mM SrfN (blue) titration with 0.3 mg/mL (green, 0.7 mM disaccharide equivalent, approximately 7∶1 ligand/SrfN dimer molar ratio), 0.8 mg/ml (orange, 1.8 mM disaccharide equivalent, approximate ratio 20∶1), and 1.8 mg/mL (red, 4.0 mM disaccharide equivalent, approximate ratio 40∶1) chondroitin sulfate polysaccharide.
(PDF)
