# Supplementary figures and images for: Structural and Functional Characterization of DUF1471 Domains of Salmonella Proteins SrfN, YdgH/SssB, and YahO
Source: PLoS One. 2014 Jul 10;9(7):e101787. doi: 10.1371/journal.pone.0101787 (PMC4092069; doi:10.1371/journal.pone.0101787)

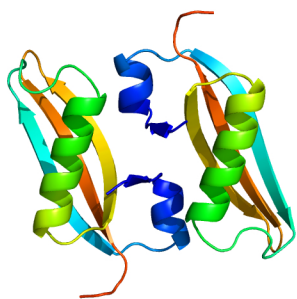

SrfN

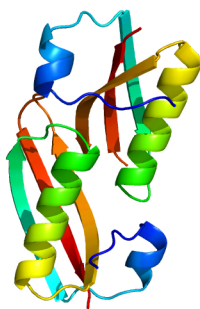

SssB-III

Supplement: Figure S1 — Comparison of solution-state SrfN dimer with crystallographic dimer for SssB-III. Ribbon cartoon depictions of SrfN dimer (left) and SssB crystallographic dimer (right). (PDF) [file pone.0101787.s001.pdf]

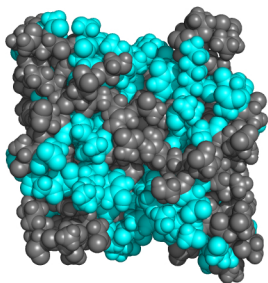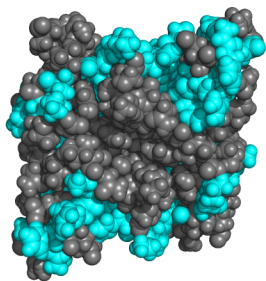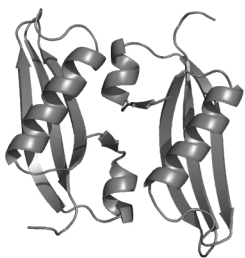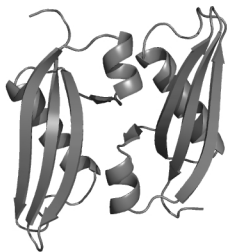

Supplement: Figure S2 — Strictly conserved residues in the SrfN subfamily. Residues conserved in all sequences shown in Fig. 1, panel C are mapped onto the structure as light blue spheres. (PDF) [file pone.0101787.s002.pdf]

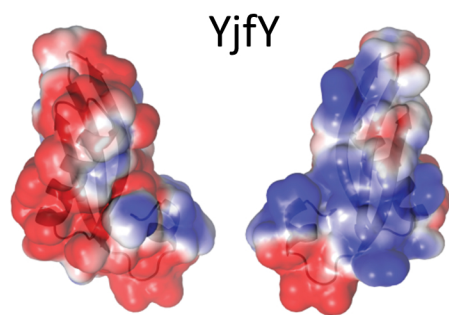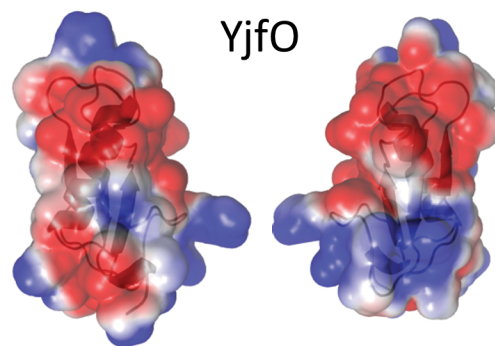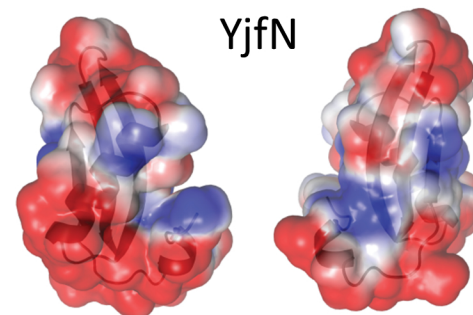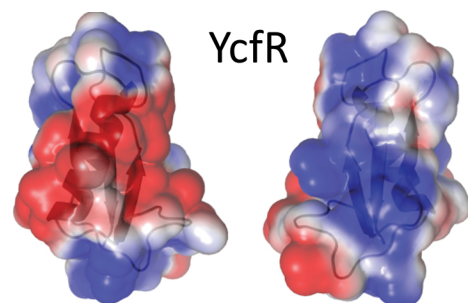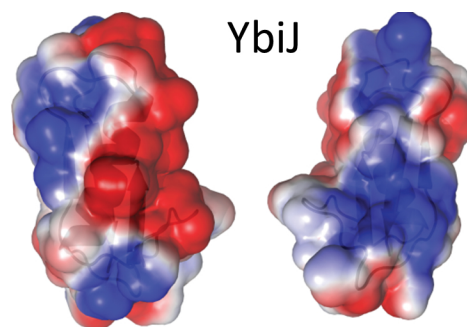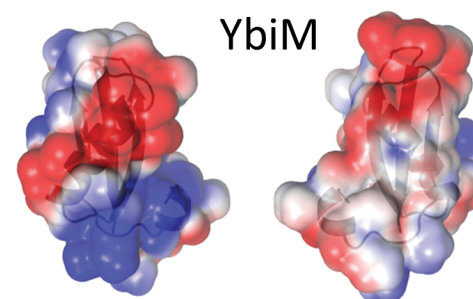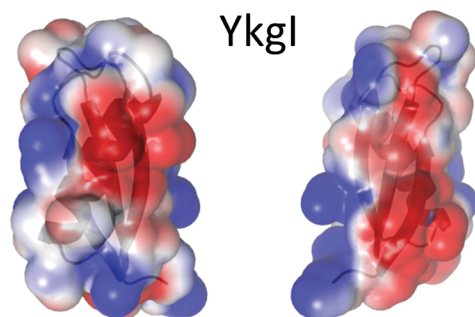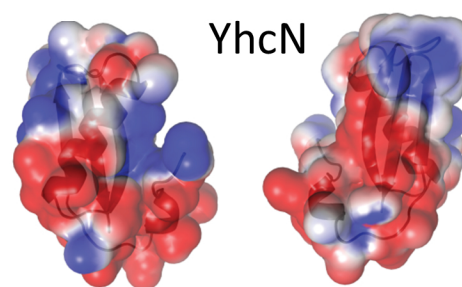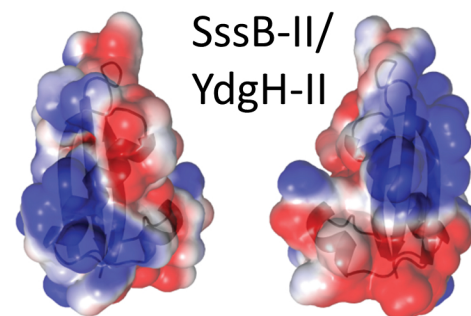

Supplement: Figure S3 — Homology models of other DUF1471 proteins from Salmonella and YbiM from E. coli . The models indicate the diversity of surface electrostatic characteristics across the family. The surfaces were calculated and are displayed in the same way described in the Fig. 2 caption. (PDF) [file pone.0101787.s003.pdf]

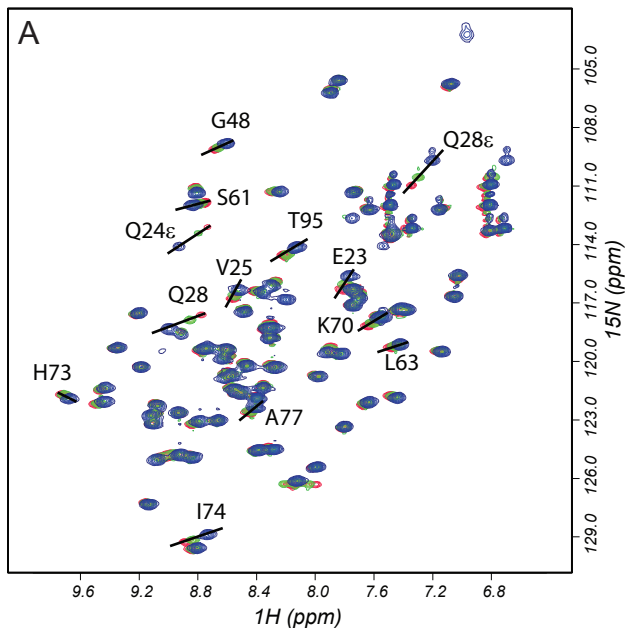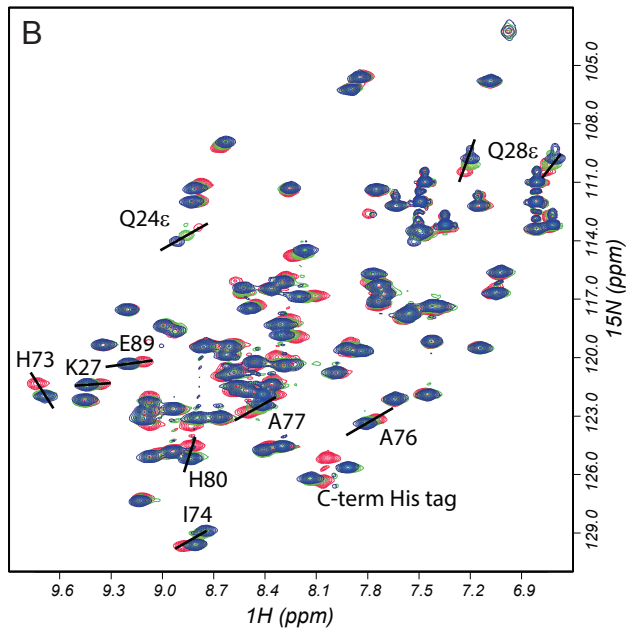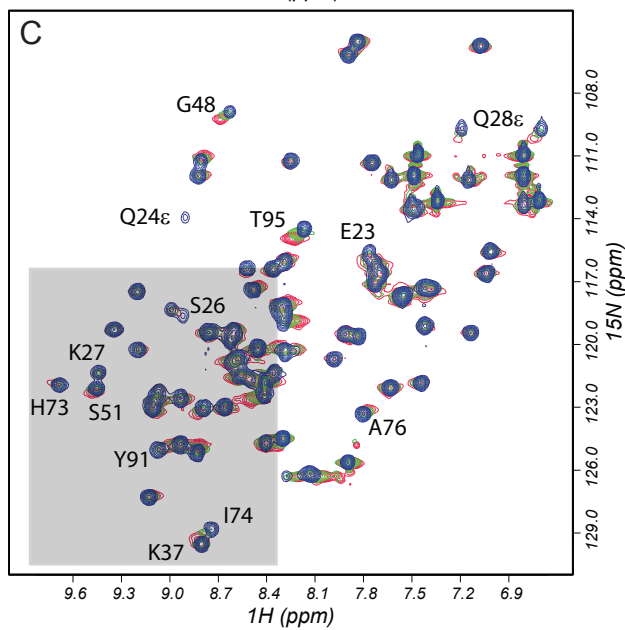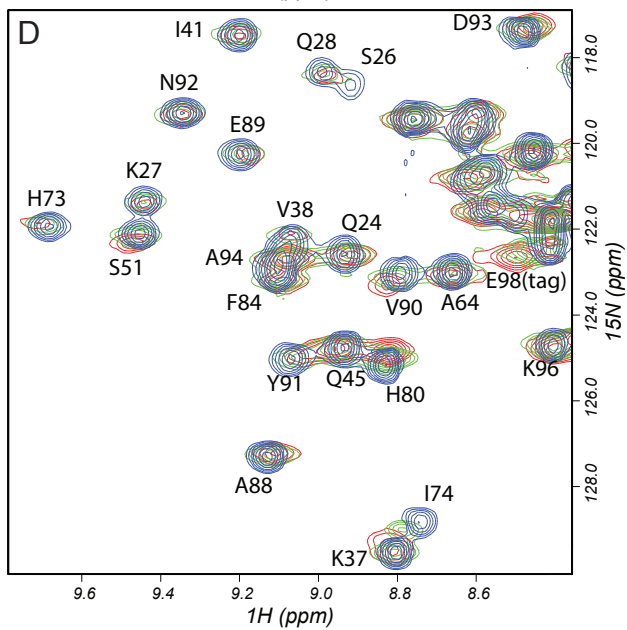

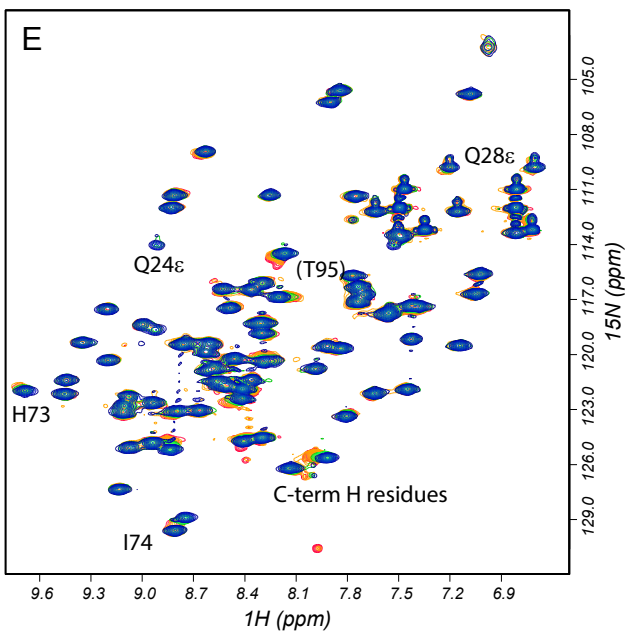

Supplement: Figure S4 — 1H-15N HSQC spectra of SrfN titrations with ligands. SrfN monomer concentrations are indicated, while the final ligand:SrfN dimer molar ratios are given in parentheses. A: 0.5 mM SfrN (blue) titration with 100 mM (green, 400∶1) and 200 mM (red, 800∶1) Na2SO4. B: 0.5 mM SrfN (blue) w/5 mM heparin disaccharide (green, 20∶1) and 4 mM hexasaccharide (red, 16∶1). C: 0.5 mM SrfN (blue) titration with 0.4 mg/mL heparin polysaccharide (green, 0.6 mM heparin disaccharide equivalent, approximately 2∶1 ligand/SrfN dimer ratio) and 2.1 mg/mL heparin polysaccharide (red, 3.1 mM disaccharide equivalent, approximate ratio 12∶1). D: expanded portion of spectrum highlighted in panel C. E: 0.2 mM SrfN (blue) titration with 0.3 mg/mL (green, 0.7 mM disaccharide equivalent, approximately 7∶1 ligand/SrfN dimer molar ratio), 0.8 mg/ml (orange, 1.8 mM disaccharide equivalent, approximate ratio 20∶1), and 1.8 mg/mL (red, 4.0 mM disaccharide equivalent, approximate ratio 40∶1) chondroitin sulfate polysaccharide. (PDF) [file pone.0101787.s004.pdf]
